# Special Notice

**Published:** 1877-05-20

**Authors:** R. C. Word

**Affiliations:** Business Manager


					﻿Editorial and Miscellaneous.
SPECIAL NOTICE.
Certain of our subscribers have order-
ed the journal on short time, and others
have been receiving it on a condition
made them in the January No. by which
they were to remit promptly on recep-
tion of February issue, pearly half the
year is gone, and they have not complied
with the engagement, subjecting us to
serious inconvenience. Friends, we are
on the cash tbasis, paying the printers
cash in advance. Your forgetfulness of
so small a matter may seem trifling to
you, but we assure you it is no light
matter with us. Please remit at once
and oblige
R. C. WORD, M. D.,
Business Manager, j
				

